# Decision quality instrument for treatment of hip and knee osteoarthritis: a psychometric evaluation

**DOI:** 10.1186/1471-2474-12-149

**Published:** 2011-07-05

**Authors:** Karen R Sepucha, Dawn Stacey, Catharine F Clay, Yuchiao Chang, Carol Cosenza, Geoffrey Dervin, Janet Dorrwachter, Sandra Feibelmann, Jeffrey N Katz, Stephen A Kearing, Henrik Malchau, Monica Taljaard, Ivan Tomek, Peter Tugwell, Carrie A Levin

**Affiliations:** 1 General Medicine Division, Massachusetts General Hospital (MGH), Harvard Medical School (HMS), Boston, MA, USA; 2 Clinical Epidemiology Program, Ottawa Hospital Research Institute (OHRI) and Faculty of Health Sciences, University of Ottawa (U of O), Ottawa, Canada; 3 Center for Shared Decision Making, Dartmouth-Hitchcock Medical Center, Lebanon NH USA; 4 General Medicine Division, MGH, HMS, Boston, MA, USA; 5 Center for Survey Research, University of Massachusetts Boston, MA USA; 6 Division of Orthopaedic Surgery, The Ottawa Hospital and U of O, Ottawa, Canada; 7 Department of Orthopedic Surgery, MGH Boston MA, USA; 8 General Medicine Division, MGH Boston MA, USA; 9 Department of Orthopedic Surgery and Division of Rheumatology, Immunology and Allergy, Brigham and Women's Hospital, HMS, Boston, MA USA; 10 The Dartmouth Institute for Health Policy and Clinical Practice, Dartmouth Medical School (DMS), Lebanon, NH, USA; 11 Department of Orthopedic Surgery, MGH, HMS, Boston, MA, USA; 12 Clinical Epidemiology Program, OHRI and Department of Epidemiology and Community Medicine, U of O, Ottawa, Canada; 13 Department of Orthopedic Surgery, DMS, Lebanon, NH USA; 14 Department of Medicine, Ottawa Hospital; Senior Scientist, OHRI, Ottawa, Canada; 15 Foundation for Informed Medical Decision Making, Boston, MA USA

**Keywords:** shared decision making, patient centered care, quality measurement, osteoarthritis, total joint replacement, decision quality

## Abstract

**Background:**

A high quality decision requires that patients who meet clinical criteria for surgery are informed about the options (including non-surgical alternatives) and receive treatments that match their goals. The aim of this study was to evaluate the psychometric properties and clinical sensibility of a patient self report instrument, to measure the quality of decisions about total joint replacement for knee or hip osteoarthritis.

**Methods:**

The performance of the Hip/Knee Osteoarthritis Decision Quality Instrument (HK-DQI) was evaluated in two samples: (1) a cross-sectional mail survey with 489 patients and 77 providers (study 1); and (2) a randomized controlled trial of a patient decision aid with 138 osteoarthritis patients considering total joint replacement (study 2). The HK-DQI results in two scores. Knowledge items are summed to create a total knowledge score, and a set of goals and concerns are used in a logistic regression model to develop a concordance score. The concordance score measures the proportion of patients whose treatment matched their goals. Hypotheses related to acceptability, feasibility, reliability and validity of the knowledge and concordance scores were examined.

**Results:**

In study 1, the HK-DQI was completed by 382 patients (79%) and 45 providers (58%), and in study 2 by 127 patients (92%), with low rates of missing data. The DQI-knowledge score was reproducible (ICC = 0.81) and demonstrated discriminant validity (68% decision aid vs. 54% control, and 78% providers vs. 61% patients) and content validity. The concordance score demonstrated predictive validity, as patients whose treatments were concordant with their goals had more confidence and less regret with their decision compared to those who did not.

**Conclusions:**

The HK-DQI is feasible and acceptable to patients. It can be used to assess whether patients with osteoarthritis are making informed decisions about surgery that are concordant with their goals.

## Background

The decision to undergo total joint replacement (TJR) for treatment of osteoarthritis can be difficult. The indications for surgery are not solely determined based on clinical features such as imaging or extent of disease; rather, providers need to work with their patients to consider how bothered patients are by their symptoms, and discuss their goals and concerns regarding treatment for their joint pain. Clinicians, consumers and researchers have recognized the "patient-centered" nature of these and other common preference-sensitive medical decisions and the importance of shared decision making to ensure high quality decisions [1-3].

For patient-centered care, providers need to ensure that patients are well informed and that medically appropriate treatments address patients' needs, wants and preferences [4]. Decision quality is an important indicator of patient-centered care and an outcome relevant for shared decision making [4,5]. The International Patient Decision Aids Standards reached consensus on a definition of decision quality as "the match between the chosen option and the features that matter most to the informed patient" [6]. The definition emphasizes two key imperatives, 1) patients are informed with the best available evidence and 2) there is concordance between what matters most to patients and the treatments they receive.

A comprehensive assessment of decision quality requires assessing patients' knowledge, the extent to which the treatment they receive matches their goals and concerns about surgical and non surgical treatments[2,7]. We found one published instrument that assessed knowledge of treatments for osteoarthritis [8], but none that assessed the extent to which treatments for osteoarthritis matched patients' goals. There is a lack of published, reliable measures that can be used to assess decision quality for patients with osteoarthritis.

Sound survey instruments must satisfy several criteria including strong psychometric properties (e.g., reliability and validity) and clinical sensibility (e.g., acceptability and feasibility) [9]. The purpose of this study is to examine performance of the Hip/Knee Osteoarthritis Decision Quality Instrument (HK-DQI) along these criteria using two study samples. The first study was a retrospective survey of patients who had made a treatment decision within the past two years and who were able to reflect on the outcomes of treatments (either surgical or non surgical). It also surveyed their providers. The second study used the HK-DQI in a randomized controlled trial of a patient decision aid with patients currently facing a decision about total joint replacement. These samples provide complementary data on the performance of the instrument. The retrospective patient sample provided an "experienced" sample to evaluate the items and to examine stability of the responses. The randomized trial assessed decision quality prospectively and could examine ability of the instrument to discriminate between those who had decision aid or not.

## Methods

The development of the Hip/Knee Osteoarthritis Decision Quality Instrument followed an extensive process that has been used to develop decision quality instruments for several common medical decisions [2,10]. First, there was a rigorous review of the published clinical evidence regarding treatment of osteoarthritis, and this was supplemented with findings from focus groups of patients with hip and knee osteoarthritis. A set of facts essential for making an informed decision and a set of goals salient for the decision were generated and then rated by a convenience sample of osteoarthritis patients (n = 88) and a multidisciplinary group of clinical experts (n = 51) [10]. Experts in survey research methods subsequently drafted questions for key facts and goals. Cognitive interviews were conducted with osteoarthritis patients (n = 10) where patients complete the survey while and interviewer watches, and then patients describe their understanding of the question and their answers to an interviewer. Based on the results, revisions were made the questions to improve acceptability and comprehensibility.

### Samples and procedures

#### Study 1

A survey was conducted in the U.S. with adults aged 40 years and older with hip or knee osteoarthritis who either had joint replacement surgery or had discussed surgery with their physician (and chose not to have it), within the past two years. Individuals with bilateral knee surgery, osteonecrosis, partial knee replacement, psoriatic arthritis, revision surgery, or rheumatoid arthritis, were excluded. Patients were recruited via patient registries at three academic hospitals and via newspaper ads. Eligible participants were selected for the sample to reach a minimum of 100 patients in key subgroups including type of osteoarthritis (hip or knee) and treatment (surgical or nonsurgical). Patients were mailed the HK-DQI, completed the paper instrument at home and returned it by mail. Non-respondents received a reminder phone call at two weeks and a reminder packet that included the original survey at four weeks. A subset of patients received a retest packet 4-6 weeks after completing their initial survey. A small gift (valued at $10 US) was provided with the initial mailing and for each completed survey.

The approach used to determine sample size for validity testing followed that advocated by Fowler [11]. First, we developed an analysis plan and identified key subgroups (joint (hip vs. knee) and treatment (surgical vs. non surgical)) and then estimated how large a sample was needed in order to reach minimum targets in the smaller subgroups. A minimum of 100 respondents in each subgroup ensured adequate power for these analyses (including sufficient observations to incorporate all key variables into the concordance model).

Primary care physicians, orthopedic surgeons, and nurses who treated patients recruited from each of the three sites were mailed a study packet with a $10 incentive. Phone or email reminders were sent at two weeks and a mailed reminder packet that included the original survey at four weeks. Providers at some sites received a $40 gift card for completing the survey.

#### Study 2

Adult patients with osteoarthritis of the hip or knee who met the guidelines for referral to an orthopaedic surgeon for TJR and had access to a TV with a VCR or DVD player were recruited for participation. Patients with inflammatory arthritis; a previous total joint replacement; or who were deaf, blind, cognitively impaired, or had a language barrier were excluded [12]. After signing a consent form, patients were randomized to receive either a patient decision aid on TJR or usual care. Patients allocated to the usual care group received a standard information booklet prepared by the hospital for patients undergoing joint replacement. The decision aid group received the same information booklet and a decision aid (video/DVD and booklet) titled *Treatment Choices for Knee Osteoarthritis *(©Health Dialog and Foundation for Informed Medical Decision Making, 2007). The decision aid describes osteoarthritis and the different treatment options and includes interviews with patients who discuss their experiences using surgical and non surgical approaches to managing their disease. Both groups were instructed to review the information at home and complete the HK-DQI. Approximately one week after recruitment, a research assistant telephoned participants to record the answers to the HK-DQI over the phone. The research assistant made an average of four calls to participants to complete the survey. Sample size for study 2 was calculated based on the primary outcome of wait time for surgery (Trials Registration # NCT00911638).

The study protocols were approved by the Institutional Review Boards at the participating sites. The procedures followed were in accordance with the ethical standards of the responsible committee on human experimentation (institutional and national) *and *with the Helsinki Declaration of 1975, as revised in 2000.

### Measures

The HK-DQI was administered in both studies along with general questions about demographics, treatments received and severity of osteoarthritis.

#### HK-DQI

The Hip and Knee instruments were almost identical (with the word "hip" replaced for "knee") and contained two main sections. The instrument is available from the corresponding author (ksepucha@partners.org).

1. Knowledge: 9 multiple choice and open-ended questions scored as correct or incorrect.

2. Goals and concerns: 7 items rated on an importance scale from 0 (not at all important) to 10 (extremely important).

#### Treatment preference

Assessed with a single item, "Which option was your personal preference?" with responses "Non-surgical approaches", "Surgery", or "I'm not sure".

#### Osteoarthritis severity

Patients completed 5 items from the Western Ontario McMasters University Osteoarthritis Index (WOMAC) pain subscale. The subscale has been used extensively for hip and knee osteoarthritis and scores range from 0 (least) to 20 (most) pain [13].

Study 1 included the following additional items in the patient questionnaire:

#### Top Three Goals and Concerns

Patients were asked to indicate the top three goals and concerns from those included in the HK-DQI.

#### Confidence

Assessed with a single item, "On a scale of 0 (not at all) to 10 (extremely), how confident are you that the decision about surgery was the right one for you?"

#### Regret

Assessed with a single item, "If you had the chance to do it again, would you make the same decision about surgery?" with responses of definitely yes, probably yes, not sure, probably no and definitely no.

#### Provider Measures (Study 1)

Providers completed the HK-DQI knowledge questions and two other questions to confirm content validity: 1) "Overall, how well does this set of items represent the key facts that patients should know before making a decision about surgery for [hip/knee] replacement?" with responses: Extremely well, very well, somewhat well, not at all well; and 2) "For each item please indicate whether you feel it is essential, very important or not important for patients to understand in order to be considered informed."

### Statistical Analysis

#### Item Retention and Deletion

A steering group that included experts in survey research, decision sciences and clinical experts in osteoarthritis examined items for issues such as difficulty (e.g., too easy or too hard), problematic format (e.g., multiple responses checked off when only one was expected), redundancy, and floor or ceiling effects (e.g., responses bunched at the bottom or top of the scale).

#### DQI- Knowledge Score

Each correct response received 1 point. Single items with multiple components had the individual component scores weighted equally in the total possible score of 1 for that item. The response, "I am not sure," was considered incorrect. The score was standardized by dividing the number of correct responses by the number of items, resulting in mean scores from 0% to 100%. A knowledge score was calculated for all respondents who completed at least 50% of the items.

#### DQI-Concordance Score

This approach follows that used by Barry et al (1995) to examine the extent to which patients' goals are associated with treatments [14]. We examined whether having TJR or not was associated with selected patient characteristics (e.g. age, gender), joint (knee or hip), and each of the goals in univariate analyses, (using t-tests for continuous variables and Chi-Squared tests for categorical variables) and in multivariate analysis using a logistic regression model with treatment received (surgery vs. non-surgical) as the dependent variable. The regression model generated a predicted probability of surgery for each patient. Patients with a predicted probability >0.5 and who had surgery or those with a predicted probability ≤0.5 and who did not have surgery, were classified as having treatments that "matched" their goals. This yielded a summary concordance score that indicated the percentage of patients whose decisions "matched" their goals. Higher scores indicate that more patients are receiving treatments that match their goals.

### Clinical Sensibility of the Instrument

#### Acceptability and Feasibility

Acceptability was examined using length of time to complete the instrument, which was self-reported by patients, and response rates. Feasibility was examined using rates of missing data, with any item with more than 5% missing responses considered problematic and any mode of administration (e.g. paper or phone) with consistently high missing data (average >5%) would be considered not feasible.

### Psychometric Evaluation

#### Reliability

Test-retest reliability was assessed by calculating the intraclass correlation coefficient (ICC) for the total knowledge score and for the individual goals and concerns. For the respondents in study 1, the scores were not expected to change over the four- to six-week period, so the target was to exceed 0.7 in that sample. We selected a 4 to 6 week retest period as that would be long enough that respondents would not remember their responses and short enough that they would be likely to complete the survey. Cronbach's alpha was not used as a measure of internal consistency for the knowledge score, as the set of knowledge items is not a measure of one underlying construct.

#### Validity

There is no gold standard for measuring knowledge, goals and concerns, or concordance so the following hypotheses relating to validity were examined:

#### Knowledge Score

(1) Discriminant validity: A key feature of a knowledge test is that it can discriminate among those with different levels of knowledge and can detect clinically meaningful differences in knowledge resulting from interventions. As a result, we tested hypotheses that (a) providers would have higher knowledge scores than patients and that (b) patients who had seen a decision aid would have higher knowledge than the control group, using two sample t-tests.

(2) Content validity: we examined the proportion of providers who considered the knowledge items essential or very important.

#### Concordance Score

The validity of the concordance score was examined three different ways. First, we examined the content validity of the goals which are the key inputs to the model. Next, we tested the discriminant validity of the regression model by examining whether it could distinguish between patients who stated a preference for surgery or non surgical treatments. Finally, we tested the predictive validity of the score itself by examining its relationship to decision confidence and regret.

(1) Content validity of the goals: We examined how many surgical and non-surgical patients selected each goal as one of their top three issues. If a small proportion of patients included an item (fewer than 20%) then we would consider deleting the item for low content validity.

(2) Discriminant validity: The regression model generates a predicted probability for surgery. Our hypothesis was that patients who stated a preference for surgery would have a higher predicted probability of having surgery based on the multivariate regression model, compared to those who were unsure. Moreover, those who were unsure would have a higher predicted probability than those who stated a preference for non-surgical approaches. These hypotheses were tested using ANOVA with planned comparisons.

(3) Predictive validity: The concordance score indicates the proportion of patients who received treatments that matched their goals. To test the predictive validity of the score, we hypothesized that patients who received treatments that matched those predicted by the regression model would have higher confidence (using a two sample t-test) and less regret (using a Chi squared test) than those who did not match.

#### HK-DQI Screener version

A shorter version of the instrument was evaluated that included 5 knowledge items (items indicated in Table 1). We examined discriminant validity of this version and also examined how well it correlated with the full score.

**Table 1:** Demographic characteristics of patient respondents for each study.

	Study 1	Study 2	
**Characteristic**	**Patients** **N = 382**	**Control group** **N = 66**	**Decision aid group** **N = 61**

**Gender**: Male (%)	169 (44)	27 (40.9)	25 (40.9)
**Age mean (SD)**	62.7 (9.6)	66.1 (9.49)	64.3(10.16)
**Race/ethnicity (%)**			
White	359 (95.5)	N/A	N/A
**Education **(%)			
≥ College graduate	209 (56)	40 (60.6)	39 (63.9)
Some college	94 (25.2)	N/A	N/A
High school or less	68 (18.1)	26 (39.4)	22 (36.1)
Missing	9 (2.4)	0	0
**Income (%)**			
<$30,000	78 (20.5)	5 (7.6)*	7 (11.5)*
$30,000-60,000	70 (18.3)	21 (31.8)**	18 (29.5)**
$60,000-100,000	89 (23.3)	13 (19.7)	21 (34.4)
Over $100,000	93 (24.3)	22 (33.3)	12 (19.7)
Missing	52 (13.6)	5 (7.6)	3 (4.9)
**Married/Committed relationship (%)**	255 (67.8)	42 (63.6)	38 (62.3)
**Months since decision (median, IQR)**	11 (7, 15)	Considering decision	Considering decision
**Had (or preferred) Surgery (%)**	235 (61) Had surgery	49 (74.2) Preferred surgery	39 (63.9) Preferred surgery
**Joint: Knee (%)**	201 (53)	61 (94)	59 (97)
**WOMAC Pain Score mean (SD)**	5.6 (4.6)	10.7 (4.2)	11.2 (4.0)

SD = standard deviation; N/A = not asked; FT = fulltime; IQR: interquartile range; * measured < $20,000; ** measured from $20,000; WOMAC = Western Ontario McMasters University Arthiritis Index

Analyses of study 1 data were conducted using PASW Statistics 18.0. Analyses of study 2 were conducted using SAS 9.1.

## Results

### Response Rates and Sample

The overall patient response rates were 79% and 92% for studies 1 and 2, respectively. The patient response rate for the retest survey in study 1 was 83%. Patient characteristics were similar across studies and are summarized in Table 1. Data on age and gender were available for non responders in study 1. Responders tended to be slightly older than non responders (mean age 62.7 vs. 60.4, p = 0.03). There was no difference in response rates by gender.

The provider response rate was 58%. The provider sample was on average, 50 years old and 68% were male. Forty-one percent were primary care doctors, 39% were orthopedic surgeons and the rest were nurses. The providers had been in practice a median of 21 years and saw a median of 100 patients with osteoarthritis each year.

### Item Retention and Deletion

One knowledge item (on usefulness of imaging) was deleted for being too difficult (only 43% of providers answered correctly). The patients' total knowledge scores ranged from 0-100% with no evidence of a floor or ceiling effect in either study. Although the responses for the goals and concerns also spanned the entire range (0 to 10) for each item, two had evidence of a ceiling effect. For "relieve pain," and "not be limited in what you can do," 71% and 77%, respectively, selected 10 out of 10 (or extremely important). The remaining analyses were conducted with the reduced set of 8 knowledge items and the full set of 7 goals and concerns. The knowledge items and responses are in Table 2 and the full survey instrument is available from the corresponding author.

### Acceptability and Feasibility

The response rates were high when administered by mail (79%, study 1) and by phone (92%, study 2). In study 1, respondents self-reported taking an average of 4.5 minutes to complete the HK-DQI (range 2-22 minutes). The mean number of missing responses was low when administered by mail or phone (1.6% and 0.08%, respectively for knowledge items) and (1.4% and 0.10%, respectively for the goals).

### Reliability

The retest reliability for the total knowledge score was ICC = 0.83 (95% CI 0.75 to 0.89). The retest reliability of the goals and concerns was acceptable: relieve pain (ICC = 0.81), avoid surgery (ICC = 0.80), not be limited in what you can do (ICC = 0.74), avoid over the counter medicine (ICC = 0.72), avoid prescription medicine (ICC = 0.75), and avoid costs (ICC = 0.72). One goal, avoid long recovery, had lower retest reliability (ICC = 0.55).

### Validity of DQI-Knowledge Score

(1) Discriminant validity: In study 1, the retrospective study, the mean knowledge scores were 61% (SD 21%) for patients and 78% (SD 14%) for providers. The knowledge scores discriminated significantly between providers and patients (mean difference 19%, 95%CI (13% to 25%), p < 0.001) for knee and 15%, 95%CI (9% to 21%) for hip p < 0.001). Study 2, the randomized trial, also demonstrated discriminant validity, as patients in the decision aid group were significantly more knowledgeable than those in usual care group (68% (SD 18%) versus 54% (SD 19%), mean difference 14%, 95%CI (8% to 21%), p < 0.001). Table 2 presents the distribution of responses for each knowledge item across the samples.

**Table 2:** Distribution of responses to the Hip and Knee Osteoarthritis Decision Quality Instruments for patients and providers across the two studies.

	Study 1		Study 2	
	**Patients**	**Providers**	**Control**	**Decision Aid**
**Item**	**N(%)**	**N(%)**	**N(%)**	**N(%)**

†1. Over time, without surgery, what usually happens to the pain from hip/knee osteoarthritis?				
Gets better	2 (0.5)	4 (4.4)	1 (1.5)	2 (3.3)
Stays about the same	15 (3.9)	5 (5.6)	3 (4.5)	6 (9.8)
* Gets worse	344 (90)	78 (86.7)	59 (89.4)	52 (85.2)
Not sure	17 (4.5)	2 (2.2)	3 (4.5)	1 (1.6)
Missing	2 (0.5)	1 (1.1)	0 (0)	0 (0)

2. For each of the following, please mark whether or not it can help relieve the pain of hip/knee osteoarthritis.				
a. Exercise				
*Yes	217 (56.8)	65 (72.2)	51 (77.3)	57 (93.4)
No	105 (27.5)	18 (20)	7 (10.6)	4 (6.6)
Not sure	53 (13.9)	5 (5.6)	8 (12.1)	0 (0)
Missing	6 (1.6)	2 (2.2)	0 (0)	0 (0)
b. Physical therapy				
*Yes	229 (59.9)	71 (78.8)	50 (75.8)	54 (88.5)
No	87 (22.8)	13 (14.4)	6 (9.1)	5 (8.2)
Not sure	61 (16.0)	5 (5.6)	10 (15.2)	2 (3.3)
Missing	5 (1.3)	1 (1.1)	0 (0)	0 (0)
c. Staying off the leg				
*Yes	162 (42.4)	58 (64.4)	36 (54.5)	39 (63.9)
No	150 (39.3)	23 (25.6)	22 (33.3)	19 (31.1)
Not sure	61 (16)	8 (8.9)	7 (10.6)	3 (4.9)
Missing	9 (2.3)	1 (1.1)	1 (1.5)	0 (0)
d. Over-the-counter pain medicine				
*Yes	257 (67.3)	87 (96.7)	50 (75.8)	50 (82.0)
No	70 (18.6)	0 (0)	10 (15.2)	6 (9.8)
Not sure	48 (12.6)	2 (2.2)	5 (7.6)	5 (8.2)
Missing	6 (1.6)	1 (1.1)	1 (1.5)	0 (0)

†3. After hip/knee replacement surgery, about how many months does it take most people to get back to doing their usual activities?				
Less than 1 month	2 (0.5)	1 (1.1)	1 (1.5)	0 (0)
1 to 2 months	75 (19.6)	22 (24.4)	14 (21.2)	7 (11.5)
*3 to 9 months	236 (61.8)	57 (63.3)	27 (40.9)	44 (72.1)
More than 9 months	34 (8.9)	9 (10.0)	6 (9.1)	7 (11.5)
Not sure	31 (8.1)	0 (0)	18 (27.3)	3 (4.9)
Missing	3 (0.8)	1 (1.1)	0 (0)	0 (0)

†4. About how many people who have hip/knee replacement surgery will need to have the same hip/knee replaced again in less than 15 years?				
More than half	28 (7.3)	1 (1.1)	8 (12.1)	5 (8.2)
About half	49 (12.8)	5 (5.6)	7 (10.6)	7 (11.5)
*Less than half	79 (20.7)	39 (43.3)	15 (22.7)	15 (24.6)
*Almost none	103 (27)	43 (47.8)	12 (18.2)	26 (42.6)
Not sure	115 (30)	0 (0)	24 (36.4)	8 (13.1)
Missing	4 (1.0)	2 (2.2)	0 (0)	0 (0)

†5. If 100 people have hip/knee replacement surgery, about how many will have less hip/knee pain when walking after the surgery?				
0-25	10 (2.6)	0 (0)	1 (1.5)	2 (3.3)
26-50	6 (1.6)	3 (3.3)	2 (3.0)	1 (1.6)
51-75	54 (14.1)	12 (13.3)	5 (7.6)	3 (4.9)
*76-100	251 (65.7)	72 (80.0)	38 (57.6)	47 (77.0)
Not sure	57 (14.9)	3 (0)	20 (30.3)	8 (13.1)
Missing	3 (.8)	3 (3.3)	0 (0)	0 (0)

†6. Serious complications can happen after hip/knee replacement surgery, such as death, life-threatening blood clots, infection and heart attack. Out of 100 people who have hip/knee replacement surgery, about how many will have a serious complication within the three months after the surgery?				
*Fewer than 5	164 (42.9)	62 (68.9)	29 (43.9)	45 (73.8)
5-10	64 (16.8)	18 (20.0)	7 (10.6)	5 (8.2)
11-15	20 (5.2)	9 (10.0)	3 (4.5)	1 (1.6)
More than 15	9 (2.4)	1 (1.1)	2 (3.0)	1 (1.6)
Not sure	115 (30.1)	0 (0)	25 (37.9)	9 (14.8)
Missing	10 (2.6)	0 (0)	0 (0)	0 (0)

7. For each of the following, mark whether or not it is a possible complication of hip/knee replacement surgery.				
a. Stomach ulcers				
*Yes	32 (8.4)	59 (65.6)	4 (6.1)	14 (23.0)
No	209 (54.7)	25 (27.8)	40 (60.6)	35 (57.4))
Not sure	126 (33)	5 (5.6)	22 (33.3)	12 (19.7)
Missing	15 (3.9)	1 (1.1)	0 (0)	0 (0)
b. Blood clot in the leg				
*Yes	332 (86.9)	45 (100.0)	51 (77.3)	52 (85.2)
No	7 (1.8)	0 (0)	3 (4.5)	3 (4.9)
Not sure	34 (8.9)	0 (0)	12 (18.2)	6 (9.8)
Missing	8 (2.1)	0 (0)	0 (0)	0 (0)
c. Migraine headaches				
Yes	21 (5.5)	9 (10.0)	5 (7.6)	4 (6.6)
*No	210 (55)	62 (68.9)	36 (54.5)	40 (65.6)
Not sure	135 (35.3)	18 (20.0)	25 (37.9)	17 (27.9)
Missing	16 (4.2)	1 (1.1)	0 (0)	0 (0)
d. Infection of the artificial hip/knee				
*Yes	279 (84.8)	45 (100.0)	51 (77.3)	51 (83.6)
No	9 (2.7)	0 (0)	1 (1.5)	2 (3.3)
Not sure	30 (9.1)	0 (0)	14 (21.2)	8 (13.1)
Missing	9 (2.7)	0 (0)	0 (0)	0 (0)

8. For each of the following, mark whether or not it is a possible side effect of using over-the-counter pain medicine for a long time. These include medicines you can buy without a prescription like Advil, Aleve or aspirin.				
a. Stomach ulcer				
*Yes	320 (83.8)	45 (100.0)	56 (84.8)	58 (95.1)
No	10 (2.6)	0 (0)	3 (4.5)	1 (1.6)
Not sure	40 (10.5)	0 (0)	7 (10.6)	2 (3.3)
Missing	12 (3.1)	0 (0)	0 (0)	0 (0)
b. Blood clot in the leg				
Yes	59 (15.4)	5 (5.6)	11 (16.7)	18 (29.5)
*No	171 (44.8)	81 (90.0)	29 (43.9)	32 (52.5)
Not sure	137 (35.9)	2 (2.2)	26 (39.4)	11 (18.0)
Missing	13 (3.4)	2 (2.2)	0 (0)	0 (0)
c. Migraine headaches				
Yes	42 (11)	23 (25.5)	8 (12.1)	12 (19.7)
*No	164 (42.9)	48 (53.3)	28 (42.4)	33 (54.1)
Not sure	160 (41.9)	18 (20.0)	30 (45.5)	16 (26.2)
Missing	16 (4.2)	1 (1.1)	0 (0)	0 (0)
d. Kidney problems				
*Yes	243 (73.8)	45 (100.0)	37 (56.1)	45 (73.8)
No	14 (4.2)	0 (0)	6 (9.1)	6 (9.8)
Not sure	63 (19.1)	0 (0)	23 (34.8)	10 (16.4)
Missing	9 (2.7)	0 (0)	0 (0)	0 (0)
e. Excessive bleeding				
*Yes	243 (73.8)	85 (94.4)	37 (56.1)	40 (65.6)
No	13 (3.9)	3 (3.3)	8 (12.1)	14 (23.0)
Not sure	63 (19.1)	0 (0)	21 (31.8)	7 (11.5)
Missing	9 (2.7)	1 (1.1)	0 (0)	0 (0)

Knowledge items reprinted with permission from the Hip Osteoarthritis Decision Quality Instrument and the Hip Osteoarthritis Decision Quality Instrument ©Massachusetts General Hospital, 2010.

*Indicates the correct answer.

**Providers (n = 45) completed both the hip and knee instruments and the distributions here combine their responses.

†Indicates those items included in the HK-DQI Screener version.

(2) Content validity: The majority of providers felt that the set of knowledge items covered the key facts extremely or very well (71%), confirming content validity. On average, 75% of providers considered the individual items essential for patients to know (range 50% for side effects of medicines to 92% for likelihood of serious complications after surgery).

### Validity of DQI-Concordance Score

(1) Content validity of goals: The percentage of patients selecting each goal as one of their top three varied depending on their treatment. For surgical patients, 97% selected "relieve pain" and 96% selected "not be limited in what you can do" as one of their top three issues. Those were followed by "avoid prescription medicine" (44%) and "avoid long recovery time" (28%). Many non-surgical patients also selected "relieve pain" (78%) and "not be limited in what you can do" (76%), followed by "avoid long recovery time" (59%), "avoid surgery" (41%), and "avoid prescription medicine" (24%). Two goals did not have strong content validity based on low frequency of selection: "avoid over the counter medicine" (14% and 5%) and "avoid costs" (6% and 9%) for surgical and non-surgical patients respectively.

(2) Discriminant validity: Four of the seven goals discriminated significantly between those who had surgery and those who did not have surgery (see Table 3, univariate results). Two goals remained significant predictors of surgery after controlling for joint (knee/hip) in multivariable logistic regression (see Table 3, multivariable results). Respondents who felt strongly about the importance of "not being limited in what you can do" were more likely to have surgery (OR 1.58 95%CI(1.18, 2.11), p < 0.001) and those who felt strongly about importance of "avoiding surgery" were less likely to have surgery (0.81 95%CI(0.75, 0.88), p < 0.001). The majority of patients in study 1 (73%) received treatment which matched that predicted by the regression model. Many non-surgical patients (41%) appeared to prefer surgery, an option that they may pursue in the future. Some patients who had surgery (18%) had scores that suggest a preference for non-surgical treatment.

**Table 3:** Univariate (t-test or chi-squared) and multivariable logistic regression analyses of factors associated with treatment received using data from study 1.

	Surgery	Non-surgical	Univariate	Multivariable	
**Factor**	**N = 235**	**N = 147**	**p**	**Odds ratio of surgery**	**95% CI**

Age (mean years)	62.7	62.7	0.95	1.02	0.99, 1.05
Education (% college graduate)	61.0	49.0	0.02	1.46	0.85, 2.51
Gender (% female)	55.8	56.5	0.83	1.20	0.71, 2.04
**Joint (% Knee)**	**43.8**	**66.7**	**<0.0001**	**0.35**	**0.20, 0.60**
On a scale of 0 to 10, how important is it to you to...					
Relieve pain	9.5	8.5	<0.0001	1.21	0.98, 1.48
**Not be limited in what you can do**	**9.8**	**8.9**	**<0.0001**	**1.58**	**1.18, 2.11**
**Avoid surgery**	**3.9**	**6.7**	**<0.0001**	**0.81**	**0.75, 0.88**
Avoid over the counter medicine	5.4	4.9	0.22	1.05	0.95, 1.16
Avoid prescription medicine	6.2	5.8	0.33	1.02	0.93, 1.13
Avoid long recovery time	7.5	8.1	0.03	0.94	0.85, 1.05
Avoid costs of surgery	3.7	4.2	0.24	0.98	0.91, 1.06

Factors that are bolded were significant in the regression model and were used to calculate the concordance score.

The regression model of treatment received had acceptable predictive accuracy (c-statistic = 0.81, Table 3). The predicted probability generated by the model also discriminated well among patients who stated a preference for surgery, those who were unsure, and those who preferred non-surgical approaches. Patients who stated a treatment preference for surgery had a significantly higher mean predicted probability of surgery compared to patients who were unsure (0.74 vs. 0.59, p < 0.001). Patients who were unsure had a significantly higher mean predicted probability compared to those who stated a preference for non-surgical approaches (0.59 vs. 0.40, p < 0.001).

(3) Predictive validity: Patients who were concordant (i.e. had treatment that "matched" that predicted by the regression model) had higher confidence in their decision (8.7/10 vs. 8.0/10, p = 0.03) and were more likely to definitely want to do the same thing again compared to those who had treatment that did not match that predicted by the regression model (68% vs. 52%, p = 0.01).

### HK-DQI-Screener

A shorter version, HK-DQI-screener, contained 5 knowledge items and 5 goals (see Table 2). The mean knowledge scores discriminated between patients in the decision aid group 67% (SD 21.2) compared to 51% (SD 24.9) in the usual care group (p < 0.001). The screener knowledge score demonstrated high reproducibility with the total DQI-Knowledge Score (Pearson correlation coefficients of 0.92 and 0.95 for studies 1 and 2 respectively, p < 0.001 for both).

## Discussion

The HK-DQI is specifically designed to evaluate decision quality for patients considering total joint replacement. The HK-DQI results in two scores, a total knowledge score that can be used to assess the extent to which patients are informed and a concordance score that can be used to assess the extent to which a group of patients received treatments that match their goals. The instrument meets many of the criteria for high quality, patient reported surveys. The instrument is acceptable to patients based on high response rates. It is also feasible to implement either over the phone or by the patient alone with few missing items and takes about 5 minutes to complete. It can discriminate between those who have different levels of knowledge and has high content validity, as both providers and patients felt it covered content that is essential for decisions about treatment of osteoarthritis. The concordance score discriminated among patients who preferred different treatment options. In addition, patients who received treatment that "matched" their goals had more confidence and less regret about their decision.

The results can be used to generate guidelines for what level of knowledge test score is needed for "informed" patients. A reasonable approach would be to use the mean score of patients who have viewed a decision aid (68% or higher) as a target. The difference between patients in the decision aid group compared to the usual care group of 14.1 points out of 100 is similar to that found in the Cochrane Systematic Review's meta-analysis of knowledge test scores of the 55 randomized controlled trials evaluating patient decision aids of 15.18 (95% CI: 11.66, 18.69) for the same comparison groups [15]. Alternatively, clinicians may use the knowledge instrument for quality improvement efforts, where the goal would be to measure patients' understanding of the key facts during the decision making process and increase scores over time.

The concordance score provides a means of measuring the extent to which treatment for osteoarthritis is tailored to patients' goals. Two goals remained significant predictors in the multivariate model, how patients felt about improving their functional status and their concern about having surgery. Often, the appropriateness of treatments like TJR is determined by severity of standard patient reported outcomes such as pain, symptom or functional status. Studies show that patients vary widely in their response to same level of symptoms [14]. This finding underscores the importance of assessing the level of bother for their symptoms, also referred to as patients' utilities, in addition to symptom severity. Patients who are very bothered by their pain or functional status should be more likely to have surgery (even if the level of symptoms is moderate). And, those who are very bothered by the prospect of surgery, should be less likely to have it (even if their level of symptoms are severe). A study of more than 2,000 men and women with osteoarthritis found that "willingness to consider replacement" was the biggest predictor of time to knee replacement, almost five times more influential than the pain score [16].

The concordance score presented here needs to be interpreted carefully. Patients were reporting their goals about one year after the decision, and the assessment might have differed closer to the time of decision making. Research on patients' decision making has repeatedly found that preferences can change with information, with experience, and they can change over time [17-19]. The regression model generates population level weights for the two goals in order to best predict treatment, and individuals may have different weightings. Patients in our study emphasized importance of additional issues, and other studies have shown that patients do consider other issues including pain, recovery time, and costs when deciding about TJR [20-22]. Despite these limitations, the regression model was able to discriminate well among patients who stated different treatment preferences supporting the validity of the concordance score. Additional research to understand whether and how patients' goals change over time, and to examine generalizability of the concordance model is needed.

There are two main ways that we envision the HK-DQI being used. The first is as a means to audit the quality of decisions for hospitals or orthopedic practices. For this purpose, patients who have recently made decisions about treatment of osteoarthritis (surgical and non-surgical approaches) may be surveyed to assess their level of understanding and their goals. To increase the practicality of the survey, a sample of patients might be surveyed periodically, e.g., all patients who had consulted a specialist about joint replacement surgery in the past six months. Study 1, that surveyed patients who had recently made a decision, replicated this type of sampling. The results of the survey can be used to determine patients' understanding of their options, and to document the extent to which treatments are tailored to patients' goals. An earlier version of these items was used this way in a national study of decision making, the DECISIONS study, which found fairly low knowledge and variable patient participation in decisions about surgery for osteoarthritis [23].

The HK-DQI (or the shorter screener version) could also be used in clinical practice as a screening tool. Patients could be surveyed in advance of a clinic visit to identify knowledge gaps and to elicit goals for incorporation into treatment recommendations [7]. This type of use requires significant integration into clinic processes to get the survey to appropriate patients in advance of the visit. For organizations that have adopted health information technology to collect patient reported outcomes, adding questions to assess patients' knowledge and their goals and concerns may be fairly straightforward. This approach can also be tied to provision of decision support, such as patient decision aids, as is happening at a few hospitals across the country [24]. Surgeons who are provided with patients' responses to the HK-DQI may find that their encounter with a patient is more efficient, allowing discussion to address gaps in knowledge and to examine stated goals and patient concerns.

A more systematic approach to documenting patients' knowledge and goals may result in improvements in care. Mancuso found that patients had many expectations about joint replacement surgery, and only 43% of patients had all their expectations completely met [25]. Further, if providers and organizations have a means of documenting that decisions are based on patients' informed values for outcomes of options, then they will be able to demonstrate that they are meeting standards for informed consent as well as new priorities set out in the healthcare reform legislation in the U.S. [4,26,27].

These two studies provide complementary evidence on the applicability of the HK-DQI for different populations (United States and Canada) and at different time points (for patients about one year after the decision and for patients currently facing the decision). However, there are several limitations to be considered. Data are lacking that would enable generalizability of the HK-DQI for non-White populations and those with limited literacy. Further, the knowledge items cover knowledge of key facts, but do not test higher level comprehension of the information. As mentioned earlier, study 1 was retrospective and may not accurately reflect patients' knowledge or goals at the time of the decision. Study 2, which did survey patients at the time of decision making, did not have sufficient number of patients selecting non-surgical options to be able to use those data to construct the concordance model. In future research, it will be important to validate the concordance model with a sample of patients facing the decision.

## Conclusions

In summary, the HK-DQI is a patient self-reported survey instrument that can be used to measure the quality of decisions about total joint replacement for knee or hip osteoarthritis. It results in two scores, a knowledge score to measure how informed patients are and a concordance score to indicate the extent to which patients received treatments that matched their goals. The instrument fills an important gap in the literature and may contribute to efforts to measure shared decision making and the delivery of patient-centered care.

## Competing interests

Drs. Sepucha, Stacey and Dr. Katz receive salary and research support from the Foundation for Informed Medical Decision Making. Dr. Levin receives salary support as Research Director for the Foundation for Informed Medical Decision Making, a not-for-profit (501 (c) 3) private foundation (http://www.informedmedicaldecisions.org). The Foundation develops content for patient education programs. The Foundation has an arrangement with a for-profit company, Health Dialog, to co-produce these programs. The programs are used as part of the decision support and disease management services Health Dialog provides to consumers through health care organizations and employers.

## Authors' contributions

All authors contributed substantially to the study including (1) the conception and design of the study (KS, DS, JK, CL), acquisition of data (SF, KC, GD, JD, JK, SK, HM, IT, PT), or analysis and interpretation of data (YC, MT, DS, KS, CC, CL) (2) drafting the article or revising it critically for important intellectual content (all authors) (3) final approval of the version to be submitted (all authors). Two authors, K.S. (ksepucha@partners.org) and D.S. (dstacey@uottawa.ca), are responsible for the integrity of the work as a whole.

## Pre-publication history

The pre-publication history for this paper can be accessed here:

http://www.biomedcentral.com/1471-2474/12/149/prepub
